# The crucial role of multimodality ultrasound in the postoperative evaluation of pediatric ischemic diseases

**DOI:** 10.3389/fpubh.2026.1875847

**Published:** 2026-09-14

**Authors:** Xiyan Zhong, Xingyuan Zhang, Sheng Yang, Rongchuan Ma, Xiaolan Tang, Mei Jin, Ting Gong

**Affiliations:** Department of Ultrasound, Chengdu Women's and Children's Central Hospital, School of Medicine, University of Electronic Science and Technology of China, Chengdu, China

**Keywords:** contrast-enhanced ultrasound, ischemic diseases, microvascular flow imaging, multimodality ultrasound, pediatric

## Abstract

Pediatric ischemic diseases are characterized by acute onset and rapid progression. Hence, it is essential to assess postoperative blood flow. Ultrasound, as a radiation-free imaging technique, is often employed to evaluate vascular perfusion in various organs. Nevertheless, conventional ultrasound is limited in detecting low-velocity blood flow and microvessels, and it is also markedly susceptible to motion artifacts in pediatric patients. Contrast-enhanced ultrasound (CEUS) and microvascular flow imaging (MFI) provide novel direction for these limitations. Here we reported four cases of ischemic diseases admitted to our hospital with detailed multimodality ultrasound features after surgery and follow ups, including CEUS in one case of neonatal renal vein thrombosis, CEUS in one case of splenic torsion, and CEUS combined with MFI in two cases of testicular torsion. CEUS revealed filling defect in four cases. MFI identified microvascular perfusion in the affected testis following surgical detorsion. During follow-up, atrophy was observed in the ischemic kidney and spleen. The testis without detectable blood flow by CEUS and MFI was surgically resected, while the retained ischemic testis exerted a transient detrimental effect on the contralateral testis.

## Introduction

While prompt diagnosis and early surgical intervention often restore the ischemic organs blood flow, the long-term impact remains a concern, such as focal infarction, atrophy, or organic loss. This study investigates the role of contrast-enhanced ultrasound (CEUS), microvascular flow imaging (MFI) and conventional measures in assessing blood function after surgery. Here we summarize our clinical experience from four pediatric patients with ischemic diseases, and we propose to use multimodality ultrasound as a clinical tool.

## Cases presentation

### Case 1

A female infant (gestational age 34 weeks, birth weight 2,080 g) was delivered by cesarean section for fetal distress. She was transferred to our hospital due to abdominal distension. Physical examination revealed abdominal distension, muscle guarding, and tenderness. Admission ultrasonography showed an enlarged left kidney (5.4 cm × 3.2 cm), increased parenchymal echogenicity, obscured corticomedullary differentiation, tortuous and dilated left renal vein with multiple thrombosis, increased resistance index and reversed diastolic flow in the left renal artery, and thrombosis in the inferior vena cava. Computed tomography revealed multiple filling defects within the inferior vena cava and renal veins, highly suggestive of thrombosis (Figure 1A). The left adrenal gland and left kidney demonstrated marked swelling with abnormal density changes, which was consistent with possible venous infarction. Two days later, CEUS was performed. The time-to-peak was 12 s in the left kidney and 5 s in the right kidney. A small volume of contrast agent was visualized in the left renal cortex, while the right renal cortex exhibited markedly greater contrast perfusion (Figures 1B, C). Then, anticoagulation therapy with enoxaparin was initiated. One month later, follow-up ultrasonography revealed a shrunken left kidney (3.8 cm × 1.9 cm), decreased peak systolic velocity, and reversed diastolic flow in the left renal artery. Magnetic resonance imaging indicated a small left kidney with heterogeneous signal and patchy hemorrhagic signals within it (Figure 1D). Rivaroxaban was prescribed, and the patient was followed up in the outpatient clinic. More than 4 months later, ultrasonography revealed further shrinkage of the left kidney (3.0 cm × 1.2 cm) with an indistinct corticomedullary boundary compared to the right kidney (Figures 1E, F). The peak systolic velocity of the left renal artery was 0.21 m/s, and the left renal vein was poorly visualized. An old thrombus was also identified in the hepatic segment of the inferior vena cava.

**Figure 1:**
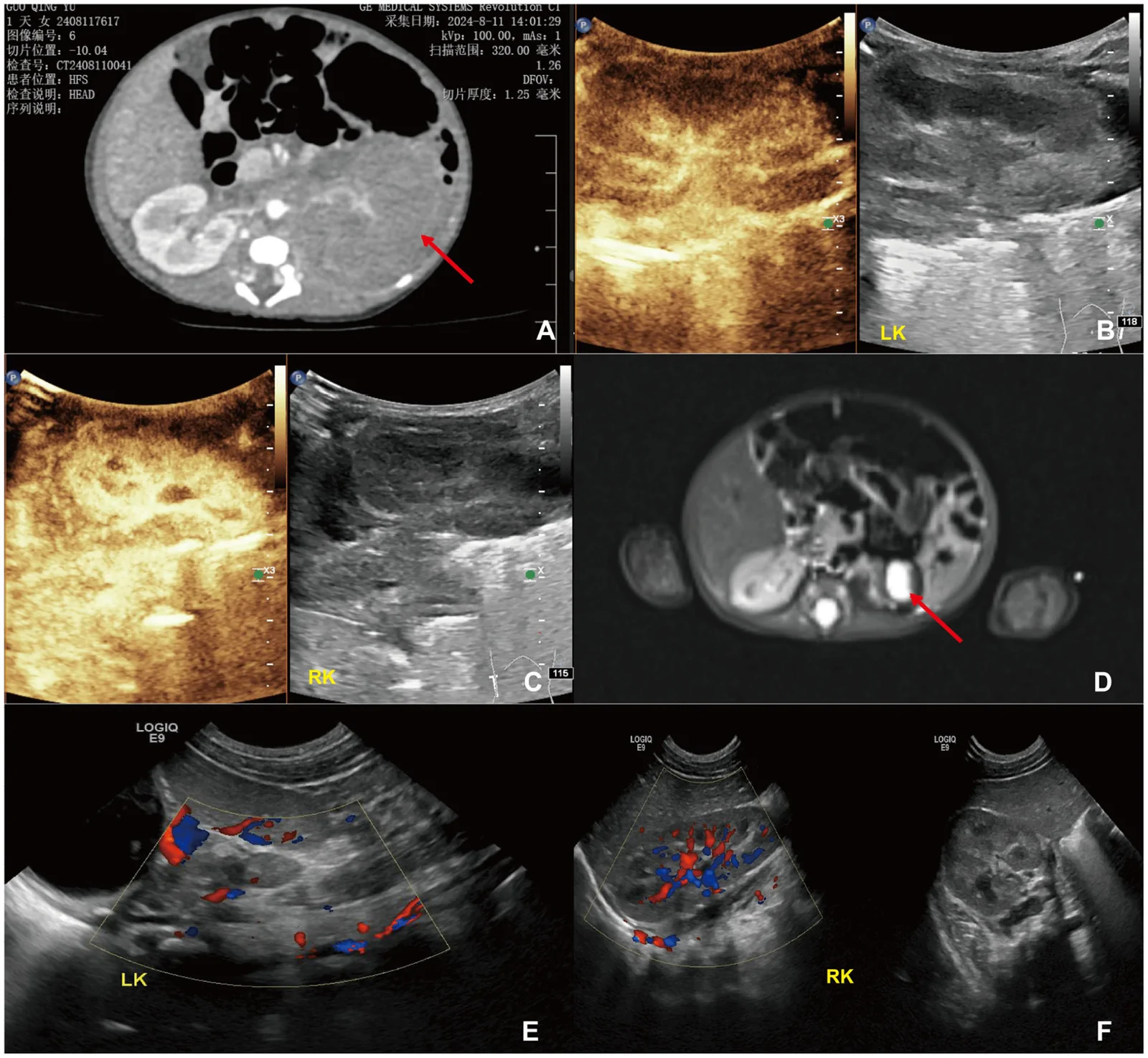
Imaging findings of Case 1. **(A)** CT image showed an enlarged left kidney (arrow). **(B)** CEUS image of the left kidney. **(C)** CEUS image of the right kidney. **(D)** MRI image showed a small left kidney (arrow) after 1 month later. **(E)** Color Doppler ultrasound image of the left kidney obtained 4 months later. **(F)** Color Doppler ultrasound and gray-scale image of the right kidney obtained 4 months later.

### Case 2

A 2-year-old female child was admitted with a 3-day history of vomiting and a 2-day history of abdominal pain and distension. Physical examination revealed diffuse abdominal tenderness. CT findings indicated splenic infarction and possible splenic artery embolism (Figure 2A). Ultrasound showed an enlarged spleen with an intercostal thickness of 3.4 cm. Multiple hypoechoic areas were observed in the parenchyma, the largest measuring approximately 4.1 cm × 2.3 cm, with ill-defined borders and irregular morphology, and no detectable blood flow signal. The splenic artery showed poor filling, and the splenic vein showed reversed flow. Further history-taking revealed a fall prior to the onset of abdominal pain. The next day CEUS demonstrated poor filling in some intraparenchymal branches with filling defects, particularly in the subcapsular region (Figure 2B). Intraoperative findings revealed a dark purple spleen with counterclockwise torsion of 360°. Postoperative ultrasound showed multiple hypoechoic areas in the splenic parenchyma. At 11 days post-surgery, CEUS again showed insufficient perfusion in some intraparenchymal branches and filling defects, predominantly subcapsular (Figure 2C). Three months after surgery, follow-up CEUS examination revealed residual filling defects measuring 2.8 cm × 1.9 cm (Figure 2D). Two years later, conventional ultrasound showed that the spleen thickness was approximately 0.8 cm, irregular shape, with heterogeneous echotexture and no obvious blood flow signals.

**Figure 2:**
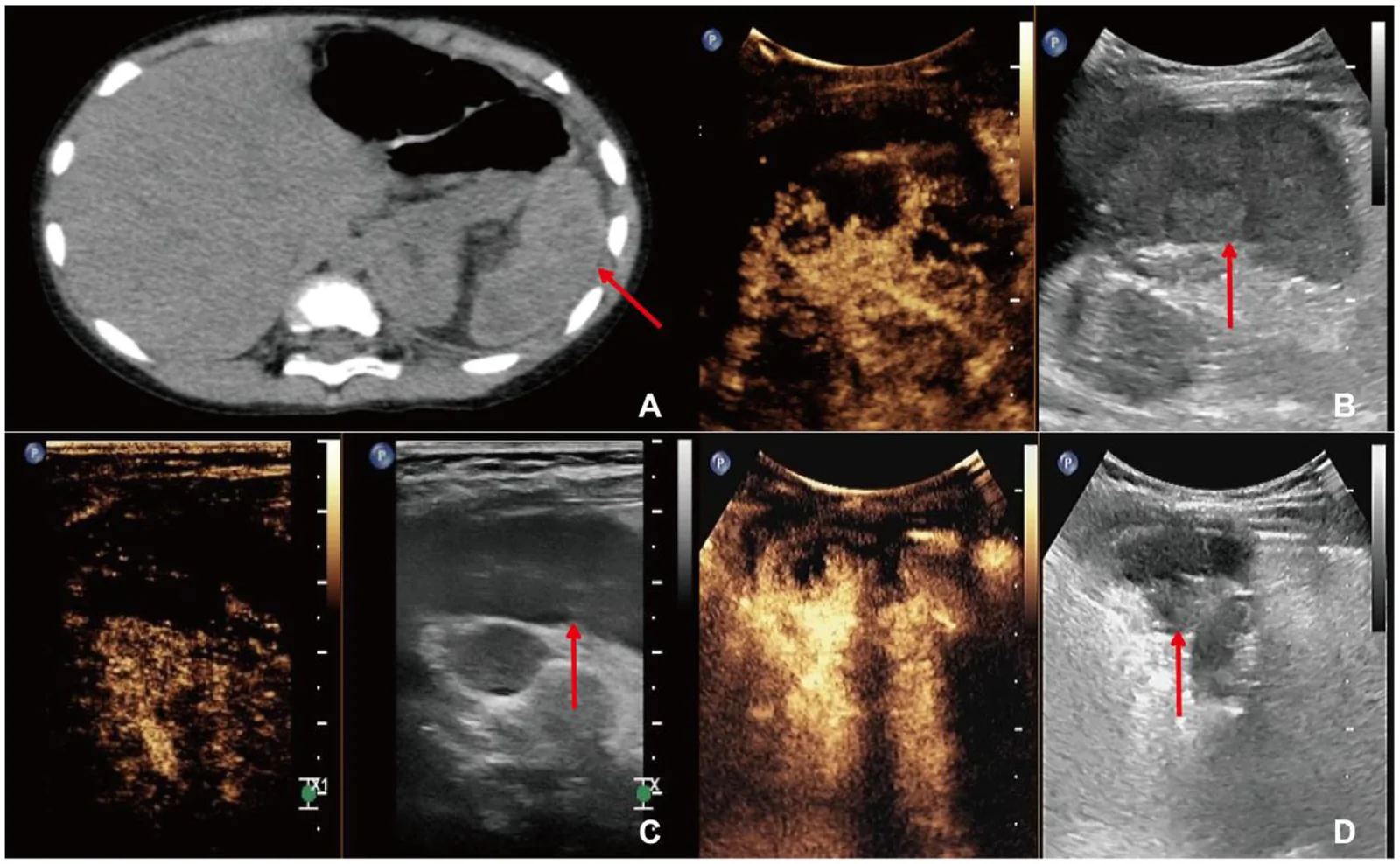
Imaging findings of Case 2. **(A)** CT image showed an enlarged spleen with a hypodense infarct (arrow). **(B)** Preoperative contrast-enhanced ultrasound image showed a filling defect area (arrow). **(C)** CEUS image at 11 days post-surgery showed a filling defect area (arrow). **(D)** CEUS image at 3 months post-surgery showing a filling defect area measuring approximately 2.8 cm × 1.9 cm (arrow).

### Case 3

A 11-year-old boy presented with 1 day history of left scrotal pain. Ultrasound showed left testicular epididymo-orchitis (Figure 3A). The left scrotum was markedly swollen, with skin redness and ecchymosis, obvious tenderness, and significant exacerbation of pain upon elevation. Intraoperatively, a 540° torsion of the left testicle was observed, the testis and epididymis appearing swollen and blackened. The color without improvement after detorsion. The family requested preservation of the left testis. One day after surgery, MFI showed reduced blood flow signals in the affected testicular parenchyma (score 1) compared to the contralateral testis (score 3). The staging criteria for MFI are as follows: 0 = absent (no color signal); 1 = poor (one internal or peripheral color spot per scan); 2 = satisfying (one internal or peripheral linear signal with multiple internal spots per scan); 3 = very satisfying (many linear signals reproducing the typical radial vascularity of the testis per scan) (1). At 7 days following surgery, MFI findings remained similar, with only starlike blood flow signals (Figures 3B, C). CEUS revealed no obvious contrast agent perfusion in the left testicular parenchyma (Figure 3D). The left testis was subsequently removed in a second surgery. One year later, follow-up ultrasound of the right testis showed no abnormalities.

**Figure 3:**
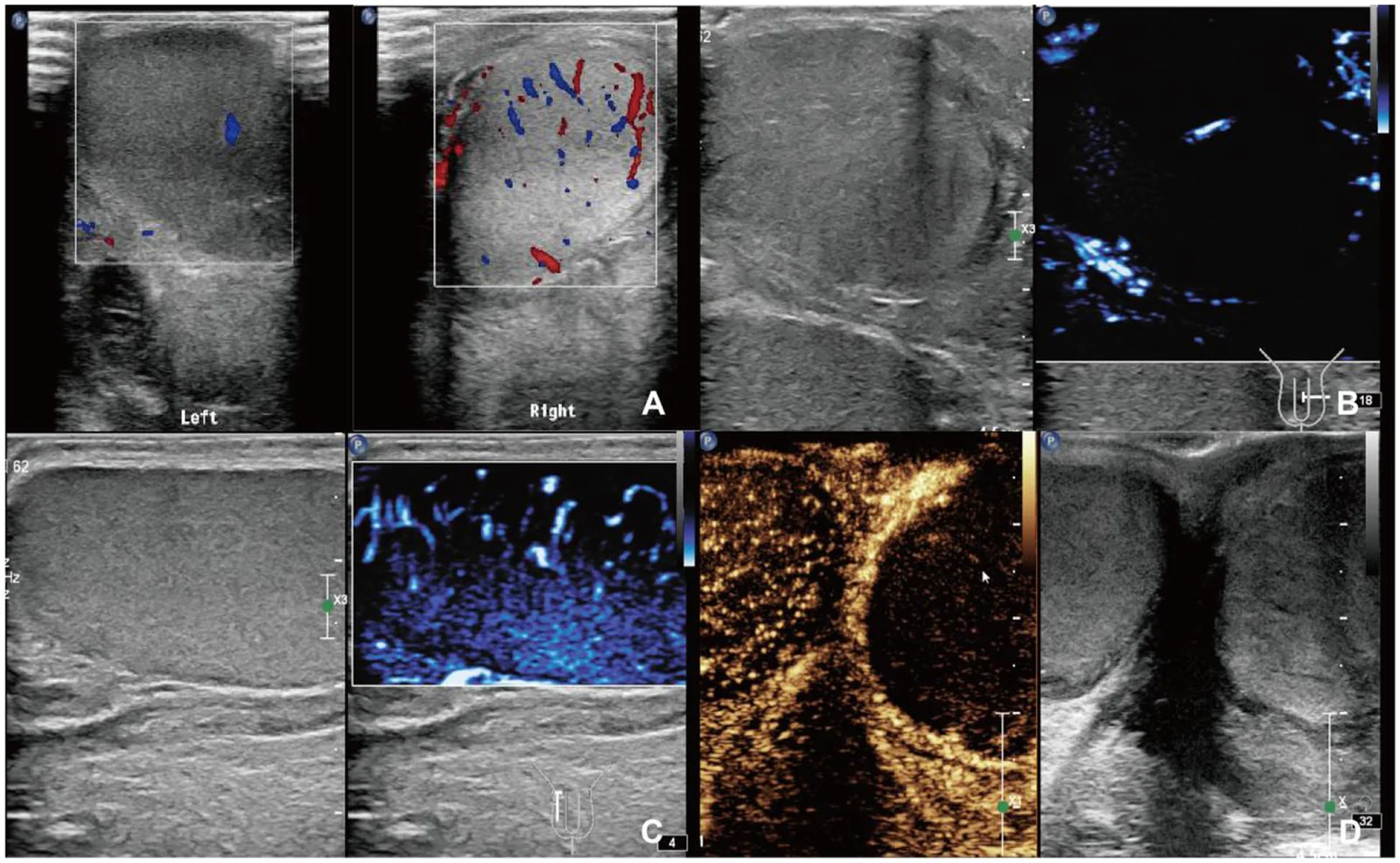
Ultrasound images of Case 3. **(A)** Preoperative testicular ultrasound image. **(B)** At 7 days following surgery, MFI image of the left testis showed reduced blood flow signals. **(C)** At 7 days following surgery, MFI image of the right testis showed richer blood flow signals compared to the contralateral side. **(D)** CEUS image on postoperative day showed no obvious contrast agent perfusion in the left testicular parenchyma.

### Case 4

An 8-year-old boy presented with a 2-h history of right scrotal swelling. Physical examination revealed marked swelling of the right scrotum with high tension and tenderness. The right testis was not well palpable, and the transillumination test was positive. Ultrasound showed testicular torsion and right hydrocele (Figure 4A). Intraoperatively, the right testis was found to be rotated 180° with severe edema without black discoloration. One postoperative day, ultrasound showed that the size and echogenicity of the right testis had no significant change compared with preoperative findings, and blood supply was normal. But the left testis was enlarged compared with the preoperative state, measuring approximately 2.3 cm × 1.4 cm × 1.4 cm, with inhomogeneous parenchymal echogenicity. Two days later, conventional ultrasound showed slit-like anechoic areas in both testes with no abnormal blood flow signals, but the MFI scores were 3 on the right and 1 on the left (Figures 4B, C). CEUS demonstrated enhancement of the right testis at 13 s with heterogeneous distribution of contrast agent, while the left testis enhanced at 2 s with sparse distribution and low enhancement (Figures 4D, E). After 1month, ultrasound showed inhomogeneous echogenicity in both testes, the left testis measured approximately 2.6 cm × 1.0 cm × 1.1 cm. Two months later, ultrasound findings were negative, and the left testis measured approximately 1.9 cm × 0.9 cm × 1.0 cm (Figure 4F).

**Figure 4:**
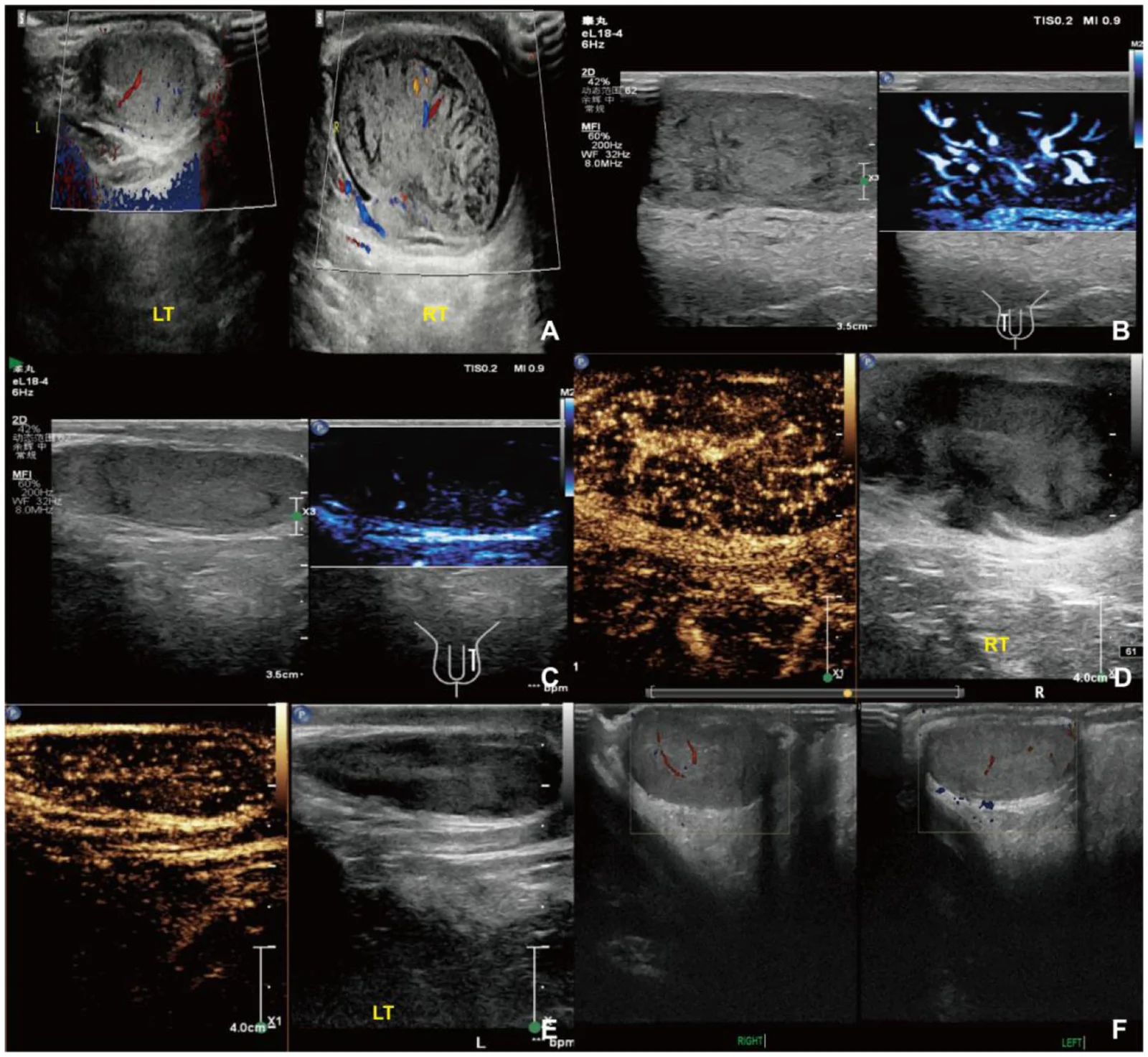
Ultrasound images of Case 4. **(A)** Preoperative color Doppler ultrasound image of the testis. **(B)** MFI image of the right testis at 2 days postoperatively. **(C)** MFI image of the left testis at 2 days postoperatively. **(D)** CEUS image of the right testis at 2 days postoperatively. **(E)** CEUS image of the left testis at 2 days post-surgery. **(F)** Color Doppler ultrasound image of the testis at 2 months postoperatively.

## Discussion

In clinical practice, the blood supply status of the organs was judged by observing the appearance and performing routine ultrasound examination. Nevertheless, these methods have limitations in detecting low-velocity blood flow. Meanwhile, uncooperative patient, operator dependence, and instrument performance that may affect Doppler sensitivity. A new Doppler module, called MFI, has recently been used to suppresses the background noise and enhance sensitivity in very weak flows (2). Beyond the microvascular imaging capability inherent to conventional Doppler modalities, this novel technique delineates microcirculation via a far more comprehensive and detailed vascular map, which depicts a greater density of thinner distal vascular branches (3). Moreover, by leveraging its background subtraction functionality, the technique markedly enhances the visibility of vascular structures (1). The use of MFI has been defined in many areas, testis being one of them. Thus, detecting microvascular flows in testicular torsion after procedure is aided (4). In recent years, CEUS has emerged as a robust diagnostic or problem-solving modality in pediatric imaging. It has been widely applied to diagnose abdominal solid organs, and its safety and efficacy have been validated (5). Compared to conventional Doppler modes, CEUS confers greater advantages in evaluating the viability of testicular parenchyma and assessing the ischemic changes in trauma patients. The distinctive merit of this technique lies in its capacity to visualize microcirculatory perfusion and detect extremely low-velocity blood flow in tiny microvascular structures.

Case 1 presented with acute kidney injury caused by acute renal vein thrombosis, an ischemic condition resulting from impaired venous drainage, and its incidence is rising annually (6). In the early stage, the left kidney was enlarged, with increased echogenicity and loss of corticomedullary differentiation. These ultrasonographic findings were consistent with the typical manifestations of moderate acute kidney injury (7). Reversed diastolic flow was detected in the left renal artery and high-resistance blood flow indicated severe renal hemodynamic compromise, which was consistent with Xu et al. finding (6). CEUS with microbubbles can provide comprehensive perfusion imaging of the kidney from the large vessels to the microvascular. Previous studies (8, 9) have demonstrated that CEUS can be exploited to assess renal perfusion and evaluate prognosis in adults. As a radiation-free imaging modality, ultrasound has long been the preferred first-line examination in pediatrics. Moreover, ultrasound contrast agents with non-nephrotoxic, CEUS can serve as an effective tool for evaluating renal perfusion in children (10). In our case, CEUS revealed that the peak time of the left kidney was longer than the right kidney. Meanwhile, long-term follow-up revealed atrophy of the left kidney.

Splenic torsion, as a rare condition, often presents with abdominal pain, along with vomiting, fever, and other nonspecific symptoms, which frequently delay diagnosis. Imaging can usually provide an accurate preoperative diagnosis. In the early stage, impaired splenic venous drainage results in splenic congestion and enlargement (11). Surgical resection is the predominant treatment modality. Among patients who undergo splenic detorsion and fixation, 32% achieve normal or congestive splenic prognosis, while 14% develop partial splenic necrosis (12). CEUS can depict non-enhancing areas throughout the spleen, enabling accurate postoperative assessment of its shape and extent (13). In our study, CEUS demonstrated a subcapsular perfusion defect in the spleen after detorsion, and there is still a partial defect 3 months later. At a 2-year follow-up, splenic atrophy was noted, with a splenic thickness of only 0.8 cm. Splenic torsion follows an arterial ischemia mechanism. The typical CEUS finding is a prolonged time to peak. However, in this case, the infarcted portion of the lesion exhibited no contrast agent entry, and no obvious delayed arterial-phase enhancement was observed compared to normal splenic parenchyma. In contrast, Case 1 represented venous ischemia. CEUS showed that the time to peak in the arterial phase was 12 s on the affected side versus 5 s on the contralateral side, and contrast signal persisted within the affected renal parenchyma during the delayed phase. Prolonged contrast washout due to obstruction of the venous outflow tract is a kinetic feature characteristic of venous ischemia.

Cases 3 and case 4 presented testicular strangulated ischemia caused by mechanical torsion. MFI is superior to conventional color Doppler flow imaging in detecting intratesticular blood flow in children with cryptorchidism and after detorsion for testicular torsion (3, 4, 14). In case 3, the MFI score was 1 after detorsion, and CEUS showed no contrast agent entry, indicating complete obstruction of the microcirculation. The affected testis was resected in a second operation, and postoperative pathology confirmed necrosis. In case 4, while a delayed time to peak on the right side (13 s) revealed by CEUS after detorsion, the MFI score was 3, and no significant abnormalities were observed on long-term follow-up. Notably, the left testis appeared to be involved during the disease course. The preoperative left testicular volume was approximately 1.836 ml, which increased to 4.508 ml on the first postoperative day, then gradually decreased, and shrank to 1.710 ml at 2 months, with the MFI score also lower than that on the right side. An animal study have also revealed diminished blood flow in the contralateral testis. This phenomenon is likely attributed to sympathetic nerve reflexes evoked by ischemia-reperfusion injury in the affected testis, which elicits vasoconstriction and further leads to ischemia and hypoxia in the contralateral tissue (15). Although conventional ultrasound shows no significant difference between the two testes after detorsion, MFI can still reveal discrepant blood flow between the two sides. Nevertheless, its impact on fertility and other outcomes requires further observation (4).

The limitations of this study include its retrospective case series and the lack of quantitative analysis of CEUS data. In addition, the sample size is relatively small. The present study primarily aims to share preliminary clinical experience and provide evidence of feasibility, rather than establish definitive prognostic criteria. Future prospective and standardized studies will be conducted to further explore the role of novel ultrasound techniques in ischemic diseases. In summary, multimodality ultrasound can provide important information on postoperative blood perfusion in ischemic diseases for clinicians, which promotes clinical diagnosis and follow-ups. Compared with conventional ultrasound, CEUS and MFI offer more comprehensive and detailed information of ischemic lesions in detecting low-velocity blood flow.

## Data Availability

The original contributions presented in the study are included in the article/supplementary material, further inquiries can be directed to the corresponding authors.
